# Mindfulness as the missing link in spiritual care: Reframing palliative care practice through presence

**DOI:** 10.1017/S1478951526103307

**Published:** 2026-08-05

**Authors:** Jeff Clyde Corpuz

**Affiliations:** Department of Theology and Religious Education, De La Salle University, Manila, Philippines

## Introduction

Spiritual care is widely recognized as a core component of holistic palliative care. Patients facing life-limiting illnesses often experience profound existential distress characterized by questions of meaning, purpose, suffering, mortality, and legacy (Miller et al. 2025). Consequently, international palliative care frameworks emphasize the importance of addressing spiritual needs alongside physical, psychological, and social concerns (Austin et al. 2025). Despite this consensus, many clinicians report feeling inadequately prepared to provide spiritual care, citing insufficient training, limited time, uncertainty about professional boundaries, and discomfort in engaging with existential questions (Nilsson 2026). As a result, spiritual care remains one of the most valued yet underdeveloped aspects of palliative practice.

While modern medicine has made remarkable advances in alleviating pain, anxiety, and other distressing symptoms, pharmacological and technical interventions alone cannot address the deeper existential dimensions of suffering encountered near the end of life. Increasing attention has therefore been given to mindfulness-based interventions (MBIs), which cultivate present-moment awareness and nonjudgmental acceptance of experience. Evidence suggests that MBIs can improve psychological well-being, reduce stress, and enhance quality of life among both patients and healthcare professionals (De Vincenzo et al. 2025). However, an important question remains insufficiently explored: beyond promoting clinician well-being, how might mindfulness influence the quality and effectiveness of spiritual care itself?

This essay argues that mindfulness is not merely a wellness strategy for healthcare professionals but a foundational clinical competency that enables effective spiritual care. By fostering presence, self-awareness, compassionate listening, and nonjudgmental engagement, mindfulness strengthens the therapeutic alliance and serves as a crucial mechanism through which evidence-based spiritual interventions achieve their effectiveness.

## Mindfulness and the foundations of spiritual care

Mindfulness refers to the intentional cultivation of awareness of present-moment experience with an attitude of openness, curiosity, and acceptance. Rather than becoming entangled in regrets about the past or anxieties about the future, individuals learn to attend to their immediate experiences without judgment (De Vincenzo et al. 2025). Extensive research demonstrates that mindfulness reduces stress, anxiety, burnout, and emotional exhaustion across diverse populations, including healthcare professionals.

In palliative care, where clinicians routinely encounter suffering, grief, and mortality, mindfulness offers benefits that extend beyond personal resilience. Narrative reviews indicate that mindfulness practices significantly reduce depersonalization, compassion fatigue, and emotional exhaustion while enhancing self-compassion and emotional regulation among palliative care providers (Miller et al. 2025). These outcomes are important not only for clinician well-being but also for the quality of patient care.

A growing body of literature suggests that mindfulness may influence how clinicians engage with patients facing existential distress. Austin et al. (2025) demonstrated that structured spiritual interventions – including dignity therapy, therapeutic life review, and meaning-centered psychotherapy – produce significant improvements in spiritual well-being, quality of life, mood, hope, and symptom burden. These interventions help patients construct meaning, affirm personal identity, reconcile relationships, and cultivate a sense of purpose despite advancing illness.

The effectiveness of these interventions cannot be attributed solely to the therapeutic protocol. Their success depends fundamentally on the quality of the clinician–patient relationship. Spiritual interventions are delivered within a relational context characterized by trust, empathy, and psychological safety. Consequently, the therapeutic alliance becomes the medium through which spiritual care exerts its healing effects. Mindfulness may therefore function as a critical but often overlooked ingredient that enhances this relational process.

## Mindfulness as the mechanism of therapeutic presence

A central feature of effective spiritual care is the clinician’s ability to be fully present with suffering. Patients nearing the end of life frequently confront fears that cannot be solved through medical expertise alone. Questions concerning death, loss, unfinished relationships, and life’s meaning often resist definitive answers (Corpuz 2026). In such moments, the clinician’s presence may be more therapeutic than any intervention offered.

Mindfulness cultivates precisely this capacity for presence. Through mindful awareness, clinicians learn to observe their own emotional reactions without becoming overwhelmed by them (Corpuz 2026; Nilsson 2026). This capacity is particularly relevant in palliative care, where encounters with mortality can activate clinicians’ personal fears, vulnerabilities, and existential concerns.

The importance of mindful presence can be understood through established frameworks for spiritual care. Effective spiritual care typically involves preparing oneself, asking meaningful questions, listening deeply, and validating the patient’s experience. Each of these dimensions is strengthened by mindfulness.

The preparatory phase requires clinicians to engage in self-reflection regarding their values, beliefs, biases, and attitudes toward death. Mindfulness develops metacognitive awareness, enabling clinicians to recognize their emotional responses and avoid projecting their personal anxieties onto patients. Such self-awareness is essential for authentic and patient-centered spiritual care.

Mindfulness also facilitates compassionate inquiry and deep listening. Clinicians often feel pressured to provide solutions, reassurance, or explanations when confronted with suffering. However, spiritual distress frequently requires accompaniment rather than resolution. Mindfulness enables clinicians to tolerate uncertainty, remain attentive during silence, and resist the impulse to prematurely alleviate discomfort through superficial responses. By remaining present without immediately seeking to fix the situation, clinicians communicate acceptance and respect for the patient’s experience.

This quality of presence strengthens the therapeutic alliance by allowing patients to feel genuinely heard and understood. In turn, patients may become more willing to explore vulnerable topics related to mortality, spirituality, forgiveness, hope, and meaning. Thus, mindfulness supports not only communication but also the relational depth necessary for effective spiritual care.

## Validation, meaning, and human dignity

Another critical dimension of spiritual care involves validating the patient’s worldview, beliefs, and existential journey. Validation does not require agreement with a patient’s spiritual perspective; rather, it requires recognition of the patient’s inherent dignity and freedom to interpret their experience according to their own values and beliefs.

Mindfulness facilitates this process by fostering a nonjudgmental stance toward diverse perspectives. Through mindful awareness, clinicians become more capable of suspending personal assumptions and engaging patients with openness and curiosity. Such an approach is especially important in contemporary healthcare environments characterized by religious, spiritual, and cultural diversity.

Patients approaching the end of life may experience acceptance, anger, despair, gratitude, peace, or spiritual struggle. Mindfulness allows clinicians to accompany patients through these varied emotional and spiritual landscapes without imposing expectations regarding how they should cope or die. By validating patients’ experiences, clinicians create a psychologically safe environment in which deeper exploration of meaning can occur.

This relational environment is essential for interventions such as dignity therapy and meaning-centered psychotherapy. These approaches invite patients to reflect on their lives, relationships, accomplishments, values, and sources of meaning. However, such reflection can occur authentically only when patients feel respected, accepted, and emotionally safe. Mindfulness contributes directly to the creation of this therapeutic space, thereby enhancing the effectiveness of evidence-based spiritual interventions (Austin et al. 2025).

## Implications for palliative care education and research

The convergence of evidence from mindfulness research and spiritual care scholarship suggests the need for a paradigm shift in palliative care education. Traditionally, mindfulness training has been promoted primarily as a strategy for reducing clinician burnout and promoting resilience. While these outcomes remain important, mindfulness should also be recognized as a core clinical competency for delivering compassionate and effective spiritual care (Nilsson 2026).

Educational programs in palliative care should therefore integrate mindfulness training not merely as a wellness initiative but as a fundamental component of professional formation. Training should include opportunities for reflective practice, contemplative exercises, self-awareness development, and experiential learning focused on presence and relational care. Such preparation can equip clinicians to engage more confidently and authentically with patients’ spiritual concerns.

At the same time, future research must move beyond examining the effects of mindfulness on clinician well-being alone. Studies are needed to investigate whether clinician mindfulness directly influences patient outcomes, including spiritual well-being, quality of life, satisfaction with care, and perceptions of the therapeutic relationship. Researchers should also explore whether mindfulness training enhances the effectiveness of established spiritual interventions such as dignity therapy and meaning-centered psychotherapy. Understanding these mechanisms would provide valuable guidance for translating evidence-based spiritual care into routine clinical practice (Miller et al. 2025; Corpuz 2026).

## Conclusion

In the context of terminal illness, clinicians cannot always offer cures, eliminate suffering, or provide definitive answers to life’s deepest existential questions. What they can offer, however, is their attentive and compassionate presence. Emerging evidence suggests that mindfulness may be the missing link connecting evidence-based spiritual interventions with meaningful patient outcomes.

Mindfulness equips clinicians with the capacity to prepare themselves, ask difficult questions, listen deeply, tolerate uncertainty, and validate the unique spiritual journeys of those facing the end of life. In doing so, it transforms spiritual care from a set of techniques into a way of being with patients in their moments of greatest vulnerability. Ultimately, spiritual care is less about doing something for patients than accompanying them with authenticity, compassion, and respect. Through mindful presence, clinicians help transform the experience of dying from one defined solely by fear and suffering into one grounded in dignity, meaning, connection, and shared humanity.
